# Levels and Determinants of Place-Of-Death Congruence in Palliative Patients: A Systematic Review

**DOI:** 10.3389/fpsyg.2021.807869

**Published:** 2022-01-13

**Authors:** Sofía García-Sanjuán, Manuel Fernández-Alcántara, Violeta Clement-Carbonell, Concepción Petra Campos-Calderón, Núria Orts-Beneito, María José Cabañero-Martínez

**Affiliations:** ^1^Department of Nursing, Alicante Institute for Health and Biomedical Research (ISABIAL), University of Alicante, Alicante, Spain; ^2^Department of Health Psychology, University of Alicante, Alicante, Spain; ^3^Department of Nursing, University of Granada, Granada, Spain; ^4^Alicante Institute for Health and Biomedical Research (ISABIAL), Alicante, Spain

**Keywords:** palliative care, congruence, place of death, systematic review, patient preference, hospital, end of life

## Abstract

**Introduction:** Congruence, understood as the agreement between the patient's preferred place of death and their actual place of death, is emerging as one of the main variables indicating the quality of end-of-life care. The aim of this research was to conduct a systematic literature review on levels and determinants of congruence in palliative patients over the period 2010–2021.

**Method:** A systematic review of the literature in the databases of PubMed, Scopus, Web of Science, PsycINFO, CINAHL, Cuiden, the Cochrane Library, CSIC Indexes, and IBECS. Information was extracted on research characteristics, congruence, and associated factors.

**Results:** A total of 30 studies were identified, mainly of retrospective observational design. The congruence values varied substantially between the various studies, ranging from 21 to 100%. The main predictors of congruence include illness-related factors (functional status, treatments and diagnosis), individual factors (age, gender, marital status, and end of life preferences), and environmental factors (place of residence, availability of health, and palliative care services).

**Conclusion:** This review, in comparison with previous studies, shows that treatment-related factors such as physical pain control, marital status, having a non-working relative, age, discussing preferred place of death with a healthcare professional, and caregiver's preference have been associated with higher levels of congruence. Depending on the study, other factors have been associated with either higher or lower congruence, such as the patient's diagnosis, gender, or place of residence. This information is useful for designing interventions aimed towards greater congruence at the end of life.

## Introduction

Place of death is a key consideration in end-of-life care. Dying in the desired or preferred place is an important consideration for patients, families and caregivers, and is therefore considered a quality indicator of palliative care (Ali et al., 2019). Historically, palliative care professionals have tried to ensure that people are cared for at home until the end of life, with dying at home being seen as an indicator of high-quality palliative care (Stajduhar and Davies, 2005). However, home is not the sole optimal option for providing quality end of life care, as other alternatives may be preferable such as hospitals, nursing homes, and hospices (Jordhøy et al., 2000; Sleeman et al., 2014). Interpreting the proportion of deaths at home as the exclusive indicator of high-quality palliative care implies that not dying at home is a failure of care and ignores the possibility that the patient may wish to die in a different place (Sekiguchi et al., 2014). For this very reason, congruence between the patient's preferred and actual place of death is increasingly being considered as a quality indicator of end of life care. As such, “congruence” is understood as the agreement between a patient's preferred place of death and actual place of death (Tang and Mccorkle, 2003). However, despite growing interest in this concept, research on the key determining factors remains scarce (Billingham and Billingham, 2013). To date, most published studies have focused on the relationship between the characteristics of the person with advanced illness at the end of life and the final place of death (Brazil et al., 2005), with widely varying results on congruence between the preferred and actual place of death (Bell et al., 2010). A clear understanding of congruence levels between the chosen and final place of death, as well as the associated variables, is critical to shaping the development of palliative care and improving end of life care (Rainsford et al., 2018; Wiggins et al., 2019).

In this context, predictors of place of death and congruence have traditionally been grouped into three categories: illness-related factors, individual factors, and environmental factors. Among the illness-related factors, the latest review and meta-analysis (Billingham and Billingham, 2013) identified cancer as having the highest congruence in final place of death (compared to other non-cancer pathologies). In terms of individual factors, ethnicity and the patient's previous preference have thus far been the variables included in earlier reviews (Bell et al., 2010). With regard to environmental factors, Bell et al. (2010) highlighted support from medical staff and being in a hospice, as well as family support.

Although the review by Bell et al. (2010) significantly advanced understanding of the factors influencing congruence, it is important to update the findings in light of subsequent research (Gomes et al., 2013; De Roo et al., 2014; De Boer et al., 2017), in which congruence between preferred and final place of death is identified as a key factor in the quality of end-of-life care. The General Medical Council's (2010) end of life best practise guidelines included the need to plan end of life care together with patients, so that members of the multidisciplinary teams caring for them can understand and address their wishes and needs improving the care to patients and families.

In light of the above, the present study aimed to conduct a systematic literature review for the years 2010–2021, following on from Bell et al. (2010) and analysing congruence levels between the preferred and actual place of death reported in the scientific literature, and the factors identified as determinants of congruence between preferred and final place of death among palliative patients.

## Materials and Methods

### Search Strategy

A systematic search was conducted to locate articles examining the congruence of place of death in patients at the end of life and predictors thereof between July 2020 and February 2021.

The keywords used to carry out the search were grouped into three main categories: terms related to “palliative care,” terms related to “patient preference” and terms synonymous with “place of death.” Searches with terms located in the title/summary and/or descriptors of the same category were joined with the Boolean connector “OR.” Once the three search categories were prepared, they were combined with the Boolean operator “AND.” Lastly, the time period (2010–2021) and language limits were applied. The final search strategy is included as Supplementary Material. The search was carried out using the following national and international databases: PubMed, Scopus, Web of Science, PsycINFO, CINAHL, Cuiden, the Cochrane Library, Information and Documentation of Science in Spain (ÍnDICEs-CSIC) and Spanish Bibliographical Index on Health Science (IBECS). This strategy was complemented by a manual search of international journals: BMJ Support Palliative, Palliative Support Care, Palliative Medicine, the Journal of Palliative Medicine and the Journal of Palliative Ageing, which address the topic of interest. The bibliographical references of the articles included in this study were also examined with a view to including potentially original studies.

### Inclusion and Exclusion Criteria

Inclusion criteria were as follows: (1) original studies analysing place of death congruence in patients at the end of life and/or its predictors, (2) studies conducted in populations over 18 years of age with both oncological and non-oncological pathologies, and (3) studies published in English, Spanish or Portuguese from 2010 onwards. Qualitative studies were excluded, as well as those that did not provide at least quantitative data on congruence in the final place of death.

### Selection of Studies

Firstly, the records obtained from the electronic searches were assessed by two independent researchers (NOB and SGS) for eligibility, based on a review of the titles and abstracts. Those studies selected in this first phase were reviewed in full text to verify that they met the established inclusion and exclusion criteria. When consensus could not be reached between the two researchers (in six of the studies) a third researcher (VCC) was consulted. At this stage the three researchers arrived at a final consensus, which is shown in the results section.

### Data Extraction

In order to facilitate data extraction, three tables were created in which the main results were synthesised. Table 1 includes information on the characteristics of the selected studies (author/s, year, research design, data collection time, sample size, sample characteristics, age, pathologies, and methodological quality assessed). Table 2 includes the main results concerned with preferred place of death and actual place of death, as well as congruence and the appearance of associated factors. Table 3 shows the main factors associated with place of death congruence, according to whether they were illness-related, individual or environmental.

**Table 1:** Study characteristics.

**Study**	**Design**	**Data collection (Months)**	**Sample size**	**Type of participants**	**Gender (% Women)**	**Age in years (SD)**	**Pathologies (%)**	**Quality STROBE/CONSORT**
1. Sheridan et al. (2021)	ROS	12	963	P	43	75.8 (NI)	Cancer: 100	20
2. Cai et al. (2020)	POS	26	290	P, CG	P: 53.8	P: 72.4 (12.38)	Cancer: 100	21
					CG: 67.2	CG: 59.3 (13.10)		
3. Skorstengaard et al. (2020)	RCT	36	N: 205	P	50	69 (NI)	Cancer: 50.1	21
			n (intervention) 102				Heart disease: 16.7	
			n (control): 103				Lung disease: 33.2	
4. Ali et al. (2019)	ROS	60	2176	P	NI	NI	Cancer: 88	18
							Non-cancer: 22	
5. Blanchard et al. (2019)	ROS	22	191	P	55.5	57.6 (13.26)	Cancer: 100	19
6. Wiggins et al. (2019)	ROS	16	1047	P	64.6	<79 (DT 13.8)	Cancer: 10.0	19
							Cardiac disease: 4.4	
							Vascular disease: 2.2	
							Respiratory disease: 1.7	
							Neurological disease: 1.6	
							Dementia: 75.8	
							Other: 4.3	
7. Bannon et al. (2018)	ROS	6	467 (363 included in the analysis)	P	60.6	<69 (57.5%)	Cancer: 100	18
8. Chiba et al. (2018)	Mixed	12	18 P/CG	P, CG, GP	P: 11.2	P: 71.9 (12.4)	Cancer: 100	18
			24 GP		CG: 94.4	CG: 61.9 (12.9)		
					GP: 16.7	GP: NI		
9. Raijmakers et al. (2018)	ROS	17	797	P	P: 53.6	P <65 57.6%	Cancer: 43.7	19
					CG: 69.3	CG <75 27.2%	Stroke: 12.4	
							COPD: 10.9	
							Heart failure: 14.6	
							Dementia: 26.5	
10. Higginson et al. (2017)	POS	17	138	P	49	74 (NI)	Cancer: 88.0	18
							Non-cancer: 12.0	
11. Lin et al. (2017)	ROS	55	481	P	39.3	70.6 (14.3)	Cancer: 70.9	21
							Non-cancer: 29.1	
12. Howell et al. (2017)	ROS	36	323	P	44.9	72.4 (12.7)	Cancer (haematological): 100	21
13. de Graaf et al. (2016)	ROS	6	130	P	52	72 (12.1)	Cancer: 89	21
							Lung failure, COPD: 3	
							Renal failure: 1	
							ALS: 2	
							Heart failure: 1	
							Dementia: 1	
							Other: 2	
14. Arnold et al. (2015)	ROS	12	1127	P	50	70 (13)	Cancer: 94	15
							Other: 6	
15. Burge et al. (2015)	ROS	24	1316 (605 included in the analysis)	P	51.3	79.1 (12.8)	Cancer: 38.1 Others: 61.9	21
16. Gage et al. (2015)	ROS	18	688	P	43.6	75.10 (NI)	NI	18
17. Ko et al. (2014)	ROS	36	695	P	43,3	≥ 65: 67.9%	Cancer: 100	19
18. Hunt et al. (2014)	ROS	7	1422	P, CG	P: 34.6	P: ≥ 60: 91.8%	Cancer: 34.6	20
					CG: 64.9	CG: ≥ 60: 55.5%	Cardiovascular disease: 24.9	
							Other: 40.5	
19. Aoun and Skett (2013)	POS	18	43 (36 included in the analysis)	P	49	74 (10.1)	Cancer: 100	20
20. Brogaard et al. (2013)	POS	21	96	P	41.7	69.9 (NI)	Cancer: 100	20
21. Fischer et al. (2013)	POS	29	458	P	35	57.9 (14.8)	Cancer: 11	18
							Other: 89	
22. Janssen et al. (2013)	POS	24	265 (206 included in the analysis)	P	35.9	67.2 (13.1)	COPD: 41.8	21
							Chronic heart failure: 29.6	
							Chronic renal failure: 28.6	
23. Capel et al. (2012)	POS	24	788	P	NI	NI	Cancer: 93	21
							Non-cancer: 7	
24. Johnson et al. (2012)	POS	15	126 (80 included in the analysis)	P	38	78 (10.7)	Chronic heart failure: 100	21
25. Abarshi et al. (2011)	ROS	12	252 (165 included in the analysis)	P	55	> 65: 80%	Cancer: 38	20
							Non-cancer: 62	
26. Alonso-Babarro et al. (2011)	POS	36	380 (228 included in the analysis)	P, CG	P: 39.5	P: 66.76 (13.4)	Cancer: 100	20
					CG: 17.9	CG: 54,32 (14.4)		
27. Escobar Pinzon et al. (2011)	CS	4	1378	P, CG	P:55.6	P: 77.6 (13.2)	Cancer: 24.2	21
					CG: 63.4	CG: 58.8 (12.8)	Dementia: 8.9	
							Cardiovascular disease: 8.4	
							Other: 12.0	
							Multimorbidity: 36.6	
							Missing/I don't know: 9.9	
28. Gerrard et al. (2011)	ROS	2007: 6	n (2007): 236	P	2007: 50.5	2007: 78 (NI)	2007: Cancer: 66	20
		2009: 6	n (2009): 275				Other: 34	
			Total: 511		2009: 42.0	2009: 72 (NI)	2009: Cancer: 76	
							Other: 24	
29. Walker et al. (2011)	ROS	24	150	P	NI	NI	NI	19
30. Holdsworth and Fisher (2010)	ROS	6	298	P	NI	NI	Cancer: 80	18
							Other: 20	

*RCT, randomised controlled trial; ROS, retrospective observational study; POS, prospective observational study; CS, cross-sectional study; COPD, chronic obstructive pulmonary disease; ALS, amyotrophic lateral sclerosis; NI, no information; RRS, rapid response service; P, patient; CG, caregivers; GP, general practitioners*.

**Table 2:** Results on preferred place, final place of death, congruence, and the existence of associated factors.

**Study**	**Preferred place of death (%)**				**Actual place of death (%)**				**Congruence**	**Associated factors**	**Percentage calculation**
	**Hospital**	**House**	**Long-term care**	**Hospice/ PCU**	**Hospital**	**House**	**Long-term care**	**Hospice/PCU**			
1. Sheridan et al. (2021)	17.7	40.6	14.1	18.1	58.0	20.0	11.0	10.2	66.1%	NI	PREF
2. Cai et al. (2020)	16.9	65.5	1.4	16.2	29.3	48.6	1.4	20.7	71.7%	1-2-3	TOT
									K: 0.527		
3. Skorstengaard et al. (2020)	GI: 1.0	GI: 33.5	GI: 3.9	GI: 2.9	GI: 17.3	GI: 42.3	GI: 0.0	GI: 34.6	GI: 52.4%	NI	TOT
	GC: 1.0	GC: 34	GC: 2.9	GC: 4.9	GC: 27.1	GC: 17.0	GC: 8.5	GC: 32.2	GC: 34.6%		
4. Ali et al. (2019)	1.2	44.8	5.5	12.0	21.2	46.5	8.4	23.8	69%	NI	PREF
5. Blanchard et al. (2019)	–	54.5	–	–	–	40.3	–	–	47.6%	1-2	TOT
									K: 0.016		
									IC 95%: (−0.107)−0.139		
6. Wiggins et al. (2019)	0.4	31.1	49.8	1.4	12.1	26.5	50.9	3.2	83.7%	1-2	TOT
7. Bannon et al. (2018)	3.5	74.7	1.7	6.0	43.7	38.1	9.0	9.2	Home: 53.4%	1-2-3	TOT
8. Chiba et al. (2018)	11.1	61.1	–	–	11.1	88.9	–	–	100%	NI	TOT
9. Raijmakers et al. (2018)	1.8	65.7	24.3	8.3	–	–	–	–	69.0%	1-2-3	TOT
10. Higginson et al. (2017)	4.0	56.0	2.0	22.0	34.0	21.0	6.0	39.0	23.0%	NI	TOT
11. Lin et al. (2017)	–	49.1	–	49.9	–	42.8	–	57.2	92.3%	2-3	TOT
12. Howell et al. (2017)	28.2	45.8	5.6	16.9	74.3	15.2	5.0	5.6	63.4%	NI	TOT
13. de Graaf et al. (2016)	–	72.0	–	21.0	6.0	70.0	–	24.0	86%	NI	TOT
14. Arnold et al. (2015)	0.7	37.0	2.4	60.0	–	–	–	–	85%	NI	PREF
15. Burge et al. (2015)^*^	15.9	73.9	10.3	–	51.5	30.4	17.9	–	51.9%	NI	PREF
									K: 0.29		
16. Gage et al. (2015)	^*^0.0	^*^76.9	^*^0.8	^*^21.1	^*^7.7	^*^63.2	^*^4.5	^*^24.7	^*^69.2%	2-3	TOT
	^^*^^*^^0.92	^^*^^*^^51.5	^^*^^*^^10.7	^^*^^*^^35.8	^^*^^*^^12.7	^^*^^*^^26.3	^^*^^*^^15.0	^^*^^*^^46.1	^^*^^*^^59.2%		
17. Ko et al. (2014)	–	100	–	–	16.7	76.0	1.3	6.0	76.0%	2-3	TOT
18. Hunt et al. (2014)	5.1	73.9	6.5	10.6	49.4	13.4	24.6	10.5	49.3%	NI	TOT
									K: 0.034		
19. Aoun and Skett (2013)	8.0	56.0	–	25.0	22.0	14.0	–	56.0	41.2%	NI	PREF
20. Brogaard et al. (2013)	3.0	45.0	1.0	16.0	26.0	41.0	6.0	26.0	44.0%	NI	TOT
21. Fischer et al. (2013)	10.0	75.0	6.0	4.0	35.0	31.0	20.0	12.0	37.0%	2	TOT
22. Janssen et al. (2013)	33.3	51.5	–	–	57.5	27.3	–	–	39.4%	NI	TOT
									K: 0.07		
23. Capel et al. (2012)	1.7	48.2	5.2	14.4	30.0	36.0	6.3	27.1	Home: 69%	NI	TOT
									Hospital: 85.7%		
									Long-term: 82.9%		
									Hospice: 81.6%		
24. Johnson et al. (2012)	*n* = 4	*n* = 69	–	*N* = 12	*N* = 41	*N* = 35	–	*N* = 21	61%	NI	PREF
25. Abarshi et al. (2011)	–	–	–	–	28.6	43.7	21.4	6.3	No deaths identified in recent days: 21%	NI	PREF
									Deaths identified in recent days 79%		
26. Alonso-Babarro et al. (2011)	–	80	–	–	–	72.4	–	–	89%	NI	PREF
27. Escobar Pinzon et al. (2011)	0.4	50.5	1.3	1.5	39.3	38.2	13.4	7.5	58.9%	2-3	PREF
									K: 0.14		
28. Gerrard et al. (2011)	+9.0	+44.0	+11.0	+36.0	–	–	–	–	76.0%	NI	PREF
	++31.0	++24.0	++7.0	++38.0							
29. Walker et al. (2011)	–	78.6	–	21.4	27.0	35.0	11.0	27.0	85.7%	NI	PREF
30. Holdsworth and Fisher (2010)	0.7	26.8	1.3	9.7	17.8	36.6	8.4	37.2	61.7%	NI	TOT
									K: 0.38		

*K, kappa; 95% CI, 95% confidence interval for cohen's kappa; GI, group intervention; CG, control group; NI, no information; PREF, calculated on those showing a preference; TOT, calculated on the total sample*;

* *users rapid response service*;

** *non-users rapid response services; +2007 data; ++2009 data; 1: illness-related factors, 2: individual factors, 3: environmental factors*.

**Table 3:** Factors associated with congruence between preferred and final place of death.

**Study**	**Factors associated with congruence between preferred and final place of death**		
	**Illness**	**Individual**	**Environmental**
Cai et al. (2020)	• Patient functional status- OR: 1.02; IC 95%: 1.01–1.04	• Civil status (divorced, separated or widowed) - OR: 0.45; IC 95%: 0.36–0.56	• Intensity of home-based nursing visits+ OR: 1.02; IC 95%: 1.00–1.04 • Hours of personal support care+ OR: 1.09; IC 95%: 1.01–1.18
Blanchard et al. (2019)	• Use of morphine + OR: 1.87; IC 95%: 1.04–3.36	• Aged + OR: 1.03; IC 95%: 1.00–1.05 • Preference to die at home - OR: 0.44; IC 95%: 0.24–0.82	
Wiggins et al. (2019)	• Patient functional status (impairment) + OR: 1.82; IC 95%: 1.06–3.13 • Ceiling of treatment of symptomatic relief only+ OR <0.2.65; IC 95%: 1.37–5.14 • Cancer diagnosis - OR: 0.52; IC 95%: 0.28–0.97)	• Cardiopulmonary resuscitation preference- OR: 0.32; IC 95%: 0.16–0.62 • Early POD register (51–250 days)- OR: 0.60; IC 95%: 0.38–0.94	
Bannon et al. (2018)	• Being unconscious during final week of life - OR: 0.1; CI 95%: 0.0–0.4	• Discussing POD with a HCP + OR: 4.7; IC 95%: 1.9–11.5 • Age (older than 80/younger than 70)- OR: 0.5; CI 95%: 0.2–1.0 • Being Presbyterian - OR: 0.30; IC 95%: 0.11–0.87	• Living in an affluent area + OR: 4.0; 95% IC 95%: 1.4–11.8 • Satisfactory care at home from a nurse + OR: 6.1; IC 95%: 2.5–15.2. • Caregiver preference of place of death (home vs. others) + OR: 17.7; IC 95%: 5.3–59.3
Raijmakers et al. (2018)	• Dementia diagnosis **+** OR: 3.33; IC 95%: 1.01–11.00 • Interaction effect: patients with dementia x preference to die at home - OR: 0.14; IC 95%; 0.04–0.56 • Stroke diagnosis - OR: 0.51; IC 95%: 0.26–0.98	• Having a partner+ OR: 2.03; IC 95%: 1.23–3.35 • PPOD Home - OR: 0.05; IC 95%: 0.02–0.12	• Have had contact with a general practitioner in the last week before death + OR: 3.85; IC 95%: 1.38–10.78 • Interaction effect with preference to die at home + OR: 6.48; IC 95%: 2.01–20.92 • High continuity of care + OR: 4.83; IC 95%: 2.36–9.89
Lin et al. (2017)		• PPOD Inpatient Hospice + OR: 17.37; IC 95%: 5.13–58.82	• Use of a High Intensity Hospice information system (HIS) + OR: 3.85; IC 95%: 1.19–12.40
Gage et al. (2015)		• Declare an initial PPOD Care Home + OR: 7.7; IC 95%: 2.5–23.4 • PPOD Own home - OR: 0.55; IC 95%; 0.3–0.8	• User of RRS+ OR: 2.1; IC 95%; 1.4–3.0 • Live with a carer + OR: 1.5; IC 95%; 1.0–2.2 • Number of days in the study - OR: 0.98; IC 95%: 0.98–0.99 • Place of residence (area 3) - OR: 0.54; IC 95%: 0.31–0.96
Ko et al. (2014)		• Aged (65-85) + OR (Belgium): OR: 0.4; IC 95%: 0.2–0.97 • Female – OR (The Netherlands): 0.1; IC 95%: 0.04–0.4 • Decision making capacity + OR (The Netherlands): 6.7; OR 95%: 1.5–29.0	• GP provision of palliative care + OR (Belgium): 9.9; IC 95%: 3.7–26.6 OR (The Netherlands): 9.7; IC 95%: 2.3–39.9 OR (Italy): 2.6; IC 95%: 1.2–2.5 • Average number of GP contacts in the 2nd, 3rd, and 4th weeks before death + OR (Italy): 0.1; IC 95%: 0.01–0.9
Fischer et al. (2013)		• Female + OR: 3.30; IC 95%: 1.25–8.72	
Escobar Pinzon et al. (2011)		• Having a non-working relative + OR: 1.79; IC 95%: 1.16–2.76 • Respondent and deceased lived together in one common household + OR: 2.28; IC 95%: 1.57–3.32	• Living in a rural municipality + OR: 1.88; IC 95%: 1.02–3.43 • Living in a rural town + OR: 2.30; IC 95%: 1.17–4.49 • Living in a small town OR: 1.95; IC 95%: 1.04–3.68)

*+, positive association between the variables; -, inverse association between the variables; POD, place of death; PPOD, preferred place of death; HCP, health care professionals; GP, general practitioners; RRS, rapid response service*.

### Methodological Quality

The methodological quality of the selected studies was examined using the CONSORT and STROBE checklists based on study design (see Table 1). The ratings of each article included in the systematic review are available as Supplementary Material.

## Results

### Search Results

**Figure 1:**
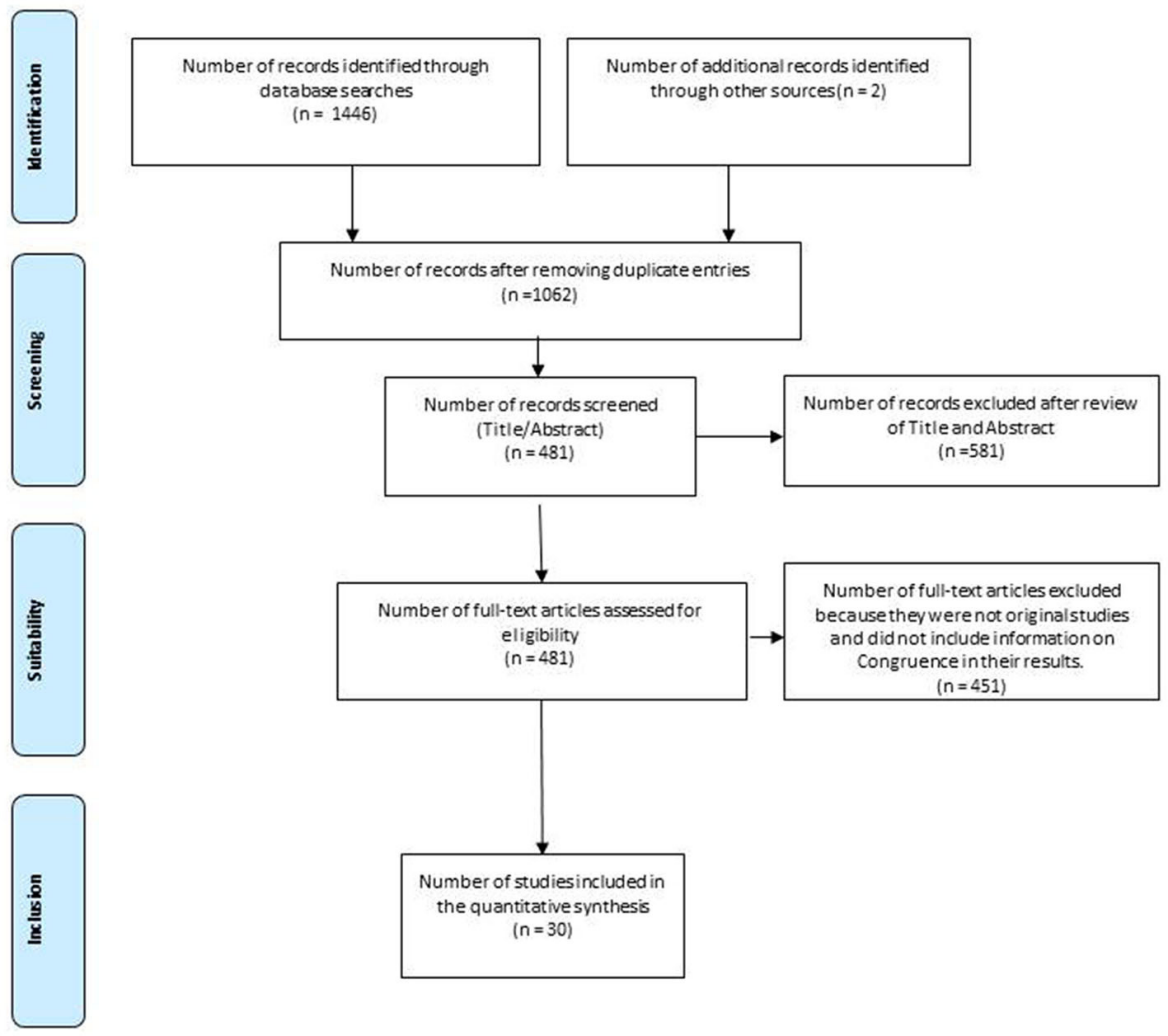
Flow diagram of the selection of studies for the systematic review. Source: Moher et al. (2009).

One thousand four hundred forty-eight articles were initially retrieved from the nine databases and the additional manual search. After eliminating duplicates, 1,062 articles were retained, and then following the initial screening process, by means of title and abstract review, 481 articles were selected and subsequently reviewed in full text. Finally, a total of 30 articles meeting the inclusion and exclusion criteria were selected and included. In this final phase, 451 articles were discarded, either because they were not original studies or because they did not record information on congruence between preferred and final place of death (see Figure 1).

### Characteristics of the Studies Included

The main characteristics of the studies in the systematic review are described in Table 1 and include: the design, the number of months of data collection, the total sample size (divided into groups when necessary), the type of participants (patients, caregivers, and/or general practitioners), the gender, age and main pathologies of the participants, and the results of the corresponding checklist. The research designs (see Table 1) were mainly retrospective observational studies (*n* = 19) and prospective observational studies (*n* = 8). Only one randomised controlled trial, one cross-sectional study and one mixed study were included. Data collection time for each study ranged from 4 (Escobar Pinzon et al., 2011) to 60 months (Ali et al., 2019). Sample sizes ranged from 18 (Chiba et al., 2018) to 2,176 individuals (Ali et al., 2019), with three studies having fewer than 100, 15 studies between 100 and 500 and 12 studies with a sample size >500 participants. Most of the studies had a sample with similar proportions of males and females. In six studies the female sample was over 60% and in four studies gender was not reported. The mean age was over 65 years in most studies (*n* = 24), and in four cases, age was not reported. A total of 10 studies exclusively analysed patients with cancer, while the rest included other chronic pathologies, or non-cancer pathologies such as dementia or stroke. In terms of the methodological quality of the studies, assessed through the different checklists, 29 of the 30 articles obtained scores greater than or equal to 18, with a range between 15 and 21.

### Preferred Place, Actual Place of Death and Congruence

Table 2 shows the percentage of preferred place of death and actual place of death in each study. In addition, the congruence value (including the kappa value where reported), the method for calculating the congruence value (using either the total sample size or only those who indicated a preference), and the analysis of associated factors is included. Preferred place of death (see Table 2) is considered first of all. A preference for hospital was reported in 23 of the 30 studies, ranging from 0% in one of the groups in the Gage et al. (2015) study to 33.3% (Janssen et al., 2013). Most of the studies (29 out of 30) reported on preference for dying at home, ranging from 24% for one of the groups reported by Gerrard et al. (2011) to 78.6% (Walker et al., 2011). Preference for dying in medium to long stay facilities was reported in 19 of the 30 studies, ranging from 0.8% (Gage et al., 2015) to 49.8% (Wiggins et al., 2019). Finally, preference for dying in a hospice or palliative care unit was reported in 23 of the studies, ranging from 1.4% (Wiggins et al., 2019) to 60% (Arnold et al., 2015).

Of the 30 studies, 27 reported some data on actual place of death, with only three providing no information at all (Gerrard et al., 2011; Arnold et al., 2015; Raijmakers et al., 2018). The percentage of people who died in hospital ranged from 6% (de Graaf et al., 2016) to 74.3% (Howell et al., 2017), and was reported in 24 of the studies. Death at home was reported in all 27 studies, ranging from 13.4% (Hunt et al., 2014) to 88.9% (Chiba et al., 2018). The percentage of deaths in medium to long stay facilities was reported in 19 of the studies, ranging from 0% (Skorstengaard et al., 2020) to 50.9% (Wiggins et al., 2019). Finally, death in a hospice or palliative care unit was reported in 22 studies, ranging from 3.2% (Wiggins et al., 2019) to 57.2% (Lin et al., 2017).

All studies reported on the congruence index. However, some studies included an overall congruence percentage while others calculated specific congruence percentages according to place of death or other variables. In 19 of the 30 studies, the congruence percentage was calculated on the total sample, while in other cases it was calculated only on those who expressed a preference, or who were part of a sub-sample (see final column in Table 2). In the 19 studies that reported overall congruence percentages, these ranged from 21% (Abarshi et al., 2011) to 100% (Chiba et al., 2018). In addition, seven studies (Holdsworth and Fisher, 2010; Escobar Pinzon et al., 2011; Janssen et al., 2013; Hunt et al., 2014; Burge et al., 2015; Blanchard et al., 2019; Cai et al., 2020) provided Kappa agreement indices, ranging from 0.016 (Blanchard et al., 2019) to 0.527 (Cai et al., 2020).

### Factors Associated With Congruence in Place of Death

Finally, the factors associated with congruence are outlined in Table 3. These factors were organised into three main categories: illness-related, individual, and environmental factors. For each of these categories, the odds ratio and their association with congruence (positive or negative) are included. Of the 30 studies included in the review, 10 reported factors that were predictors of congruence in place of death (see Table 2). Five identified illness-related factors as being risk variables (Bannon et al., 2018; Raijmakers et al., 2018; Blanchard et al., 2019; Wiggins et al., 2019; Cai et al., 2020), 10 identified individual factors (Escobar Pinzon et al., 2011; Fischer et al., 2013; Ko et al., 2014; Gage et al., 2015; Lin et al., 2017; Bannon et al., 2018; Raijmakers et al., 2018; Blanchard et al., 2019; Wiggins et al., 2019; Cai et al., 2020), while seven identified environmental factors (Escobar Pinzon et al., 2011; Ko et al., 2014; Gage et al., 2015; Lin et al., 2017; Bannon et al., 2018; Raijmakers et al., 2018; Cai et al., 2020).

Positive and negative (lack of congruence) associations were reported for the factors associated with congruence (see Table 3). In the first instance, a number of illness-related factors were identified. The patient's functional status had both a positive and negative association with congruence (Wiggins et al., 2019; Cai et al., 2020). Cancer diagnosis (Wiggins et al., 2019) and stroke (Raijmakers et al., 2018) were negatively associated with congruence, while dementia (Raijmakers et al., 2018) was positively associated. Treatment-related factors, such as morphine use (Blanchard et al., 2019) and ceiling of treatment of symptomatic relief (Wiggins et al., 2019), were positively associated with congruence. Finally, the patient's level of consciousness during the final few days was negatively associated with congruence (Bannon et al., 2018).

Secondly, with respect to individual factors, marital status (being in a relationship) was positively associated with congruence in three studies (Escobar Pinzon et al., 2011; Raijmakers et al., 2018; Cai et al., 2020), as were having a non-working relative (Escobar Pinzon et al., 2011) and age (Ko et al., 2014; Bannon et al., 2018; Blanchard et al., 2019). Significant variability was found in relation to preference for place of death. Preference for dying at home (Gage et al., 2015; Raijmakers et al., 2018; Blanchard et al., 2019), preference for cardiopulmonary resuscitation, and early registration of place of death (Wiggins et al., 2019) were negatively associated with congruence. Preference for dying in a hospice (Lin et al., 2017), having discussed preferred place of death with a healthcare professional (Bannon et al., 2018), and having decision-making capacity (Ko et al., 2014) were positively associated with congruence. Other variables had no clear association, for instance being female, which was both positively (Fischer et al., 2013) and negatively (Ko et al., 2014) associated with congruence in two of the reviewed studies. Finally, one study looked at religious belief and found that being Presbyterian (as opposed to other religions) was negatively associated with congruence (Bannon et al., 2018).

Finally, environmental factors such as place of residence (Escobar Pinzon et al., 2011; Gage et al., 2015; Bannon et al., 2018) and indicators of adequate care by nurses, medical staff, or a caregiver were associated with higher congruence (Ko et al., 2014; Gage et al., 2015; Bannon et al., 2018; Raijmakers et al., 2018; Cai et al., 2020). Other variables associated with higher levels of congruence include the family's role, the caregiver's own preference for place of death (Bannon et al., 2018), being in a high intensity hospice (Lin et al., 2017), using a Rapid Response System (RRS) (Gage et al., 2015), and access to palliative care (Ko et al., 2014). Only the amount of time spent in the study (assessed in number of days) and one of the places of residence in the Gage et al. (2015) study had a negative association with congruence.

## Discussion

The present study conducted a systematic review of congruence values between preferred and actual place of death, as well as the main determinants of this congruence in the period of 2010–2021. The results of the present review analysing a total of 30 studies with over 14,000 participants indicate considerable variability in the congruence values identified, in line with results identified in previous reviews (Bell et al., 2010; Billingham and Billingham, 2013).

One of the most important objectives of palliative care is to enhance the quality of life of terminally ill patients and their environment (World Health Organization, 2021). It is therefore necessary to encourage health services to involve patients and their families in the decision-making process about their treatment and end of life care, and it is vitally important to know the patient's preferred place of death and to make it easier for them to die there (Baik et al., 2019). The results of the present review show that, although home remains one of the preferred places of death, previous studies indicate that hospitals are often one of the main places where death occurs (Nilsson et al., 2017). On this note, a recent systematic review and meta-analysis in cancer patients shows a high degree of variability in the preferred place of death where, while home remained the highest with 55% preference, other participants preferred hospital (17%) and hospice (10%) (Fereidouni et al., 2021). In the data obtained in the present review, home appears as the preferred place of death in all the studies that evaluate this, although the values for the hospital vary greatly between studies, exceeding 30% in several (Gerrard et al., 2011; Janssen et al., 2013).

With regard to congruence, in 21 of the 30 studies the percentage was below 75% for the different locations assessed, which seems to indicate that the level is still insufficient, in line with previous studies (Billingham and Billingham, 2013; Howell et al., 2017; Baik et al., 2019). It is therefore important to consider which factors and variables influence congruence levels, as has been identified in numerous studies in the case of place of death (Cabañero-Martínez et al., 2020).

The factors predicting the congruence rate in this review include those related to the illness itself, and individual and environmental factors. Since Bell et al.'s (2010) systematic review, several new factors influencing congruence have been identified (see Table 4). Treatment-related factors associated with physical pain control, marital status, having a non-working relative, age, discussing preferred place of death with a healthcare professional, and caregiver's preference have been associated with higher levels of congruence. Depending on the study, other factors have been associated with higher or lower congruence such as the patient's diagnosis, gender, or place of residence.

**Table 4:** Summary of the factors affecting congruence.

**Enhancing congruence**	**Decreasing congruence**
**Illness-related**	**Illness-related**
Patient's functional status	Patient's functional status
Dementia	Cancer
Treatment-related aspects	Stroke
	Patient's level of consciousness during last days
**Individual**	**Individual**
Marital status	Preference for dying at home
Non-working relative	Preference for cardiopulmonary resuscitation
Age	Early registering of place of death
Preference for dying in a hospice	Being female
Having spoken to a healthcare professional about the preferred place of death	Religious beliefs
Decision-making capacity	
Being female	
**Environmental**	**Environmental**
Place of residence	Place of residence
Indicators of adequate care	Time of participation in the study
Family role	
Caregiver's preference	
High intensity hospice	
Having palliative care	

In terms of disease-related factors, the use of morphine and other pain-related treatments were positively associated with congruence between the preferred and actual place of death. As such, both variables may refer to the existence of a palliative intervention, which in many cases will be associated with the patient's end of life preferences having been explored, discussed, understood and taken into account (Saugo et al., 2008; Scaccabarozzi et al., 2017). However, the patient's own poorer state of health and unconsciousness at the end of life were negatively associated with congruence in place of death. This could be due to the fact that, in these cases, the doctor and family are the main responsible for the decision-making process and may not be aware of the patient's own preferences, if the issue has not been addressed beforehand, or that these preferences have been put to one side (Medina et al., 2012). In addition, specific diagnoses such as cancer or stroke were negatively associated with congruence in the studies evaluated. In the first case, one possible explanation is that cancer patients in advanced stages of the disease, despite greater knowledge of their prognosis, may still die in hospital due to the wider treatment options available (Fereidouni et al., 2021). Furthermore, this may also indicate an important shortcoming with respect to effective home care plans, since their effectiveness, a priori, should make it possible for many cancer patients to die in their preferred place (Cabañero-Martínez et al., 2020). Previous reviews have highlighted an increased risk of incongruence in non-cancer pathologies, although they also noted that there appeared to be no correlation between overall levels of congruence and the percentage of patients with cancer (Billingham and Billingham, 2013). In the case of stroke, several studies have pointed to the difficulty professionals have in identifying patients' palliative needs, as well as being able to communicate adequately regarding end of life preferences (Eriksson et al., 2016; Cowey et al., 2021).

In terms of individual factors, being married or in a relationship, as well as living with a partner, was positively associated with congruence in the preferred and actual place of death. In this regard, it should be noted that the patient's decision is usually respected by their spouse or closest relatives, particularly when there is an advance directives document (Agulles Simó, 2010; Landa and García, 2017; Bejarano Gómez et al., 2019). This document enables patients to exercise their right to plan and decide on their active and palliative treatment guidelines once they are unable to make decisions (Mira et al., 2010). Previous studies suggest that patients who had prepared advance directives received care that was strongly associated with their preferences, increasing the likelihood that these plans would be implemented (Leff et al., 2000; Silveira et al., 2010; Halpern et al., 2020). Therefore, achieving congruence between the preferred place and final place of death should be an aspect that is reflected in the advance directives of those at the end of their lives. However, it is important that healthcare professionals have knowledge, training and experience in the use of advance directives in order to integrate them into end of life decision making (Aguilar-Sánchez et al., 2018).

Other factors related to individual variables refer to end of life preferences, such as the choice not to perform cardiopulmonary resuscitation, and informing healthcare staff of the patient's preferences or decision-making capacity. In this respect, the associations found positively relate these factors to congruence between place of preference and final place of death. These results are in line with previous research where adequate communication with health workers and the patient's decision-making capacity are associated with high levels of congruence (Burge et al., 2015; Cohen et al., 2015; Finucane et al., 2019). Other factors such as gender and age have been shown to be predictors, albeit with positive values in some cases and negative values in others.

Finally, in relation to environmental factors, higher levels of congruence have been found when the family's choice coincided with that of the patient in line with previous studies (Raziee et al., 2017; Cai et al., 2021). In addition, variables directly related to the availability of health services and palliative care have also been positively associated with congruence in many of the studies included in this review. Furthermore, some studies have highlighted the importance of the neighbourhood, area or size of the city in which the patient lived (Escobar Pinzon et al., 2011; Gage et al., 2015). Recent studies have shown how variables related to socio-economic status can have an important influence on the place of death. Nolasco et al. (2020) noted that the probability of dying in hospital, compared to dying at home, is higher as the level of economic deprivation in the urban area of residence increases, both for palliative care-related illnesses and for other pathologies. Future studies should clarify the role that such variables can play in predicting congruence levels in different pathologies.

The review findings suggest that little has changed regarding the congruence percentages identified in the literature. Despite the evolution of palliative care and the importance given to patients' preferences for end of life care, a high percentage do not die where they wish to. Factors such as the provision of palliative care, the role of healthcare professionals, family dynamics, and adequate care are important for improving the level of congruence among palliative care patients. Discussing end of life preferences with both the patient and family members or caregivers may facilitate the process of dying in the preferred place. Future studies analysing the balance between patient and caregiver preferences are also needed to identify their roles in achieving congruence.

The main limitations of the studies included in this review include the use of retrospective observational designs and the lack of prospective designs with which to study congruence. In addition, basic sociodemographic information about participants (such as age, gender, and main diagnosis) is not always included, making it difficult to interpret the results. Research on non-oncological conditions and the role that the diagnosis can play in predicting congruence levels is also an important line of research to be considered (Martí-García et al., 2020). Finally, some of the factors associated with congruence have both a positive and negative association (i.e., place of residence or gender), hence further research is required in order to clarify their role.

The present study has a number of strengths and limitations. In the first instance, a high degree of heterogeneity has been identified in the congruence data whereby, while in many studies this came from the total sample, in other cases only data from the sub-samples were indicated. Secondly, there has also been a high degree of variability in the associated factors across studies, with many being assessed using a single question, or by means of continuous variables in some studies and categorical variables in others. Nonetheless, a methodological assessment of all the research selected in this review was carried out, revealing adequate values. Further studies are required to gain more in-depth knowledge about the factors influencing congruence in order to optimally plan health services and improve the quality of end of life care.

## Conclusion

In conclusion, the present review shows variability in levels of congruence between preferred and final place of death. The main predictors of congruence include illness-related factors (functional status, treatments, and diagnosis), individual factors (age, gender, marital status, and end of life preferences), and environmental factors (place of residence and availability of health, and palliative care services).

## Data Availability Statement

The original contributions presented in the study are included in the article/Supplementary Material, further inquiries can be directed to the corresponding authors.

## Author Contributions

SG-S, MF-A, VC-C, and MC-M: conceptualisation, methodology, software, validation, formal analysis, investigation, resources, data curation, visualisation, and project administration. SG-S, VC-C, CC-C, and NO-B: formal analysis. SG-S, MF-A, VC-C, CC-C, and MC-M: writing original draft preparation, writing review and editing, and funding acquisition. MF-A and MC-M: supervision. All authors contributed to the article and approved the submitted version.

## Funding

This work was supported by grants UGP-18-255, UGP-19-253, and UGP-20-038 from ISABIAL and PI17/00328 from the Carlos III Health Research Institute (ISCIII) belonging to the Spanish Ministry of Health, and by FEDER (A way of making Europe) project funds.

## Conflict of Interest

The authors declare that the research was conducted in the absence of any commercial or financial relationships that could be construed as a potential conflict of interest.

## Publisher's Note

All claims expressed in this article are solely those of the authors and do not necessarily represent those of their affiliated organizations, or those of the publisher, the editors and the reviewers. Any product that may be evaluated in this article, or claim that may be made by its manufacturer, is not guaranteed or endorsed by the publisher.
