# Supplementary material for: Levels and Determinants of Place-Of-Death Congruence in Palliative Patients: A Systematic Review
Source: Front Psychol. 2022 Jan 13;12:807869. doi: 10.3389/fpsyg.2021.807869 (PMC8792401; doi:10.3389/fpsyg.2021.807869)
Supplement: Supplementary file 1 [file Table_1.DOCX]

**Supplementary Material 1.** Search strategy for the systematic review

Search: (((“Patient Preference”[Mesh]) OR ((((choice[Title/Abstract]) OR (preference[Title/Abstract]))) OR (decision[Title/Abstract])) OR (Wish[Title/Abstract]))) AND (((“Palliative Care”[Mesh] OR “Palliative Medicine” [Mesh] OR “Hospice and Palliative Care Nursing” [Mesh]) OR (“Terminal Care” [Mesh]))) OR ((((palliative[Title/Abstract]) OR (“terminal care” [Title/Abstract])) OR (“end-of-life” [Title/Abstract])) OR (hospice[Title/Abstract])))) AND (((“Place of death” [Title/Abstract]) OR (“site of death” [Title/Abstract])) OR (“location of death” [Title/Abstract])) Filters: from 2010 – 2021 Sort by: Most Recent
